# Dopamine enhances recovery after traumatic brain injury through ubiquitylation and autophagic degradation of RIPK1

**DOI:** 10.1186/s12964-024-01515-y

**Published:** 2024-02-19

**Authors:** Hui Luo, Ning Liu, Chao Lin

**Affiliations:** Department of Neurosurgery, the First Affiliated Hospital of Nanjing Medical University, Nanjing, 210029 China

**Keywords:** Traumatic brain injury, Apoptosis, Autophagy, RIPK1, Chip

## Abstract

**Background:**

Although the neurotransmitter dopamine (DA) plays a crucial pathophysiologic role after traumatic brain injury (TBI), its function and specific underlying mechanisms of action remain unclear.

**Methods:**

Adult male mice underwent controlled cortical impact (CCI). We administered DA intraperitoneally to mice for 14 consecutive days, starting 8 h before CCI. On day 3 after brain injury, cortical lesion volume and brain water content were measured. On days 7–13, behavioral tests were performed.

**Results:**

Herein we report that DA inhibits neural death after injury, which is mediated via the dopamine D1 receptor (DRD1). Our results also showed that DRD1 signaling promotes RIPK1 ubiquitination via the E3 ubiquitin ligase Chip and then degradation through autophagy. Importantly, in vivo data revealed that DRD1 signaling prevented neural death, suppressed neuroinflammation, and restored many TBI-related functional sequelae.

**Conclusions:**

These data reveal a novel mechanism involving dopamine, and suggest that DRD1 activation positively regulates Chip-mediated ubiquitylation of RIPK1—leading to its autophagic degradation.

**Supplementary Information:**

The online version contains supplementary material available at 10.1186/s12964-024-01515-y.

## Introduction

Traumatic brain injury (TBI) is considered to be the leading cause of substantial mortality and long-term disabilities in children and young adults worldwide [1, 2]. TBI is a highly heterogeneous injury that can cause a range of long-term complications—including cognitive disorders, motor disability, chronic traumatic encephalopathy and persistent vegetative states [3–5]. Unfortunately, a specific therapy for TBI does not yet exist, and standard treatment remains supportive in nature. Following the primary mechanical injury, TBI results in delayed secondary-injury events due to metabolic, neurochemical, inflammatory, and cellular changes that account for many of the neurologic dysfunctions [3, 6].

Although neuroinflammation is widely considered to be a key secondary injury mechanism after brain injury, and it has been reported to contribute to neurologic deficits [3], the function of neuroinflammation after TBI remains controversial. Both experimental and clinical studies indicate that neuroinflammation exerts both detrimental and beneficial effects on neurologic recovery, which likely differ in the acute and delayed phases following brain injury [3, 7, 8]. To create optimal conditions for neuronal repair and regeneration after TBI, the current neuroinflammation-based treatment is to promote its neurotrophic and beneficial functions while reducing the neurotoxic and detrimental effects [9, 10].

Dopamine (DA) is a key neurotransmitter that regulates behavior, movement, neurodevelopment, endocrine systems, and emotion. DA agonists have been widely used clinically in the treatment of TBI, Parkinson disease, pituitary tumor and neuropsychonic disorders [11]. DA receptors—termed DRD1–DRD5—display, have different functions in modulating the autophagy, inflammation, and apoptosis [11, 12]. Accumulating evidence has indicated that DA behaves as an important molecule in alleviating neuroinflammation by binding to DA receptors [11–14]. Based on these observations, we hypothesized that DA may ameliorate the TBI-induced neuroinflammation.

Herein we demonstrate that DA is an endogenous regulator of neuroinflammation and neuronal apoptosis by mediating receptor interacting protein kinase 1 (RIPK1), and suggest that the DRD1 is as a potential target for the treatment of TBI.

## Materials and methods

### Patients

Human brain tissue was obtained from the tissue bank of the Institutional Review Board-approved Department of Neurosurgery at the First Affiliated Hospital of Nanjing Medical University (NMU; Nanjing China) (Supplementary Table 1). Brain tissue resected from the pericontusional area of the temporal or frontal cortices of severe TBI patients who underwent decompressive craniectomy. Brain tissue resected from the temporal cortices of epilepsy patients who underwent surgical treatment was used as a control. The human aspect of our study was approved by the Ethics Committee of the First Affiliated Hospital of NMU (2023-SR-717).

### Mice and protocols

This study encompassed male C57BL/6 mice (12 weeks of age) weighing 24 ± 5 g. *RIPK1*^−/− ^mice were obtained from the Model Animal Resource Center (MARC; Nanjing, China), beclin1 transgenic mice from the JAX lab, GFP-LC3 transgenic mice from Beijing CasGene Biotech, and *Drd1*^*−/−*^ and *Drd2*^*−/−*^mice from the lab of Dr. Zhou [14]. All transgenic mice were of C57BL/6J background. Standard laboratory animal food and water were provided by the Animal Center of NMU. Mice were maintained in a pathogen-free environment and housed on 12-h day/night cycles at an ambient temperature of 22 °C ± 2 °C.

Cortical lesion volume and brain water content were measured on day 3 after brain injury, and behavioral tests were performed on post-TBI days 7–13. Fourteen days after brain injury, mice were sacrificed and samples were obtained for other designated experiments.

### Traumatic brain injury

As previously described, controlled cortical impact (CCI) was applied to cause cerebral contusion [3]. Briefly, injury to the right cortex was performed using a 3-mm flat-tip impounder with a velocity of 6 m/s, a depth of 0.6 mm, and a duration of 100 ms.

### Primary cortical neurons and stretch injury (SI)

Primary cortical neurons were isolated from 14-day-old embryonic mouse cortex and used for designated experiments on 14 day in vitro, according to the study protocol. TBI was produced via mechanical stretch of the neurons cultured on a flexible silastic membrane using a pneumatic pressure pulse as described previously [3].

### Drug treatment

DA, A-68,930, bafilomycin A (BAF), and 3-methyladenine (3-MA) were purchased from Sigma-Aldrich (St. Louis, Missouri, USA). We administered A-68,930 intraperitoneally to mice at a dosage of 5 mg/kg/day for 14 consecutive days, starting 8 h before brain injury [15]. The dosage of DA is 50 mg/kg/day [15]. BAF and 3-MA were used according to our previous study [3]. Saline (vehicle) was used in the sham group.

### siRNA-mediated gene silences

siRNA was chemically synthesized by GenePharma (Shanghai,China).The siRNA sequences were: siDrd1 (5′-GGGAGACUAAAGUCCUGAAdTdT-3′), siDrd2 (5′-GGCCAUGCCUAUGUUGUAUdTdT-3′), siDrd3 (5′-GGUGGAGUCUGGAAUUUCAdTdT-3′), siDrd4 (5′-CCUGGAGAACCGAGACUAUdTdT-3′), siDrd5 (5′-CUGCCUAUGUCCACAUGAUdTdT-3′), siChip (5′-CCTATGACCGCAAGGAATT-3′), siFbxo10 (5′-GGATCCTTCGCGGGACGTCCT-3′), siMarch7 (5′-GGACUUAUGUAGAAUUUGUdTdT-3′).

### Lactate dehydrogenase (LDH) release assay

LDH release was done with a commercial assay kit (catalog no. ab102526, Abcam) according to the manufacturer’s instructions.

### Cortical lesion volume and brain edema

An MRI apparatus for small animals at 11.7T (Bruker, AVANCE 500WB) at the Animal Core Facility of NUM was used to measure cortical lesion volume. As previously described, we calculated lesion volume from the summation of defective areas of on each slice, and multiplied it by slice thickness. The brain water content was measured as water content: (%) = ([wet weight-dry weight]/wet weight) x 100%.

### Immunoblot analysis

The level of target protein was measured by immunoblot analysis as the previous study [3]. The follow primary antibodies were used: p62 (catalog no. ab56416, Abcam), LC3 (catalog no. 2775s, Cell Signaling Technology), beclin1 (catalog no. 3495, Cell Signaling Technology), NeuN (catalog no. ab104224, Abcam), β-actin (catalog no. 3700, Cell Signaling Technology), RIPK1 (catalog no. 3493, Cell Signaling Technology), RIPK3 (catalog no. 95,702, Cell Signaling Technology), IL-1β (catalog no. 31,202, Cell Signaling Technology), caspase-3 (catalog no. ab184787, Abcam), ub (k-48, catalog no. ab140601, Abcam), ub (k-63, catalog no. ab179434, Abcam), Chip (catalog no. ab1791, Abcam).

### Reactive oxygen species (ROS)

Dihydroethidium (DHE; catalog no. D11347; Thermo Fisher) staining was applied to assess the production of ROS in brain tissues, and samples were incubated with DHE and DAPI according to the manufacturer’s instructions. We observed the frozen slices under confocal microscopy and assessed expression with Image J (NIH, USA).

### Enzyme-linked immunosorbent assay (ELISA)

The levels of IL-1β, IL-6, and TNF-α in the brain tissues were measured by ELISA kits (Elabscience, China) according to the manufacturer’s instructions. The absorbance value was evaluated with a microplate spectrophotometer (Tecan, Switzerland).

### TUNEL staining

A terminal deoxynucleotidyl transferase dUTP nick-end labeling (TUNEL) kit was purchased from Roche (catalog no. 12,156,792,910, Roche, Germany). The mice were sacrificed and perfused with 0.1 mM PBS (pH 7.4). In brief, coronal frozen Sect. (7 μm in thickness) were incubated with TUNEL reaction mixture (red) and then stained with DAPI (blue), and the apoptosis was assessed as the number of red positive cells/DAPI-stained cells.

### Neurological severity score (NSS)

In brief, a 10-point NSS was applied to assess motor function, beam balance, and startle reflexes. One point was recorded for failing to complete each task.

### Novel object recognition (NOR)

On test day 1, mice were placed on an empty surface for 5 min; on day 2, we placed the mice on the same surface that included two identical objects for 5 min; on day 3, we placed the mice on this same surface that included an “old” object and a “new” object for 5 min. The percentage of time spent in probing the “new” object was recorded to evaluate the extent of learning and memory.

### Morris water maze (MWM)

The MWM was implemented by the same experimenter at the identical time with a tracking device and software (Chromotrack 3.0, San Diego Instruments). In a series of daily trials, we placed mice at a random locations (north, south, east, and west), and a probe trial was done 2 h after the last hidden-platform trial.

### Statistical analysis

All data are expressed as the means ± standard deviation (SD). We used the unpaired Student’s *t* test was used to compare the differences between two groups with Prism Software 6.04 (GraphPad Software, Inc.). NSS and MWM data were analyzed by two-way ANOVA for an overall statistic, followed by Tukey’s test for between-group comparisons. Significance was defined as a *P* value < 0.05.

## Results

### DA treatment rescued behavioral deficits after CCI

**Fig. 1:**
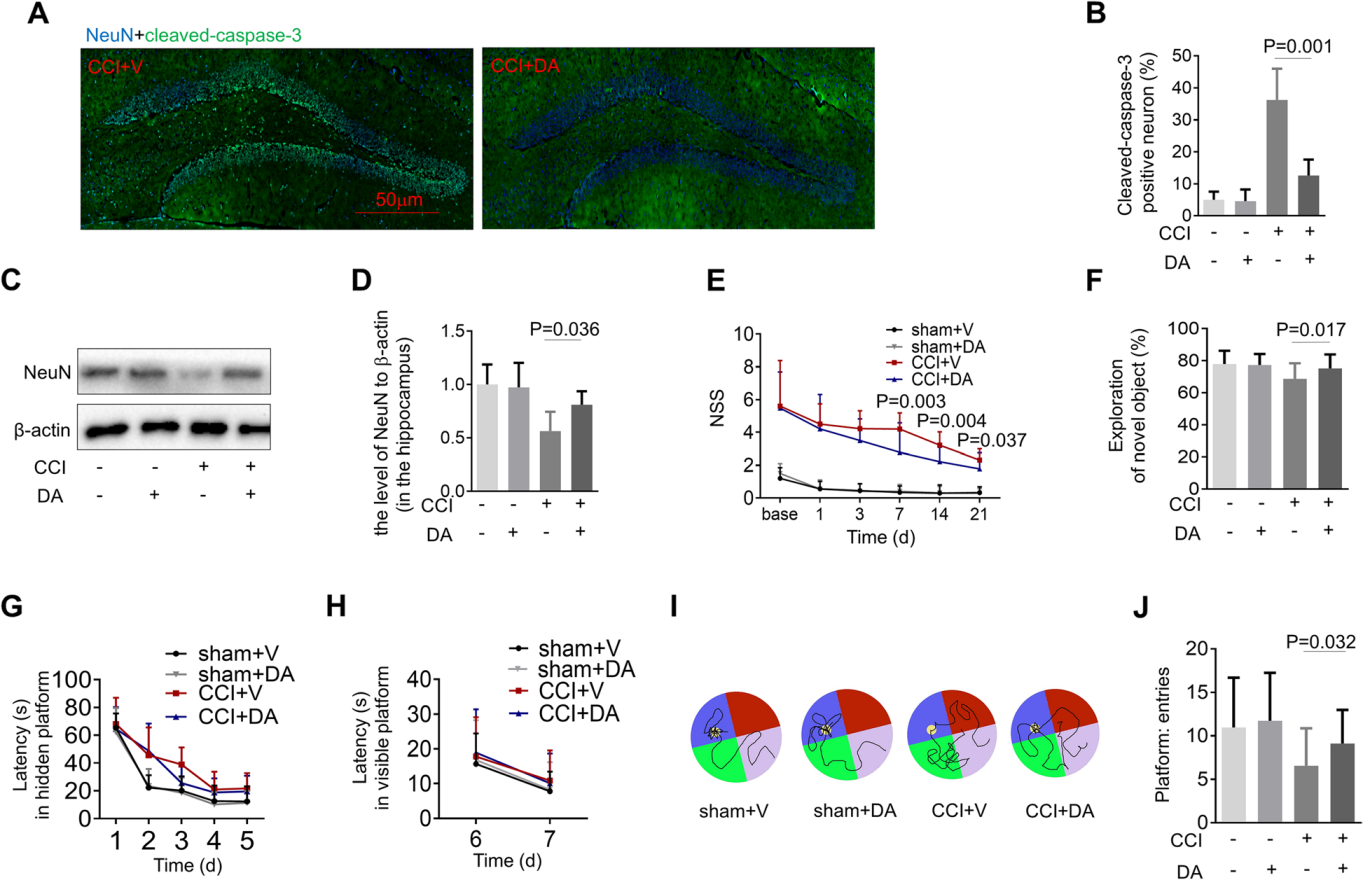
DA inhibits neural death and mitigates behavior deficits after traumatic brain injury. **A** Representative photomicrographs of NeuN (blue) and cleaved-caspase-3 (green) in the ipsilateral hippocampus (*n *= 5/group). **B** Quantification of cleaved-caspase-3 fluorescence intensity in the ipsilateral hippocampus (*n *= 5/group). **C**, **D** Immunoblotting and quantification of NeuN in the ipsilateral hippocampus. **E** Neurological severity score (NSS, between DA- and vehicle-treated mice subjected to brain injury). **F** Novel object recognition (NOR). **G**-**J** Morris water maze (MWM). Graph showing hidden platform trial (**G**) and visible platform trials (**H**). Representative images (**I**) and quantification (**J**) of probe tests. **E**-**J** *n* = 25/group. All data are mean ± SD. V, vehicle

In the present study, the enhanced hippocampal expression of cleaved caspase-3, a marker of apoptosis, was observed after CCI (Fig. 1A and B). The elevation of the cleaved caspase-3 was inhibited by DA treatment (Fig. 1A and B). Furthermore, the level of NeuN in the hippocampus was significantly increased in the CCI group treated with DA versus those treated by vehicle (Fig. 1C and D), suggesting that DA had the effect of ameliorating neural death induced by CCI.

To further determine the impact of DA on behavioral or functional outcomes after CCI, we assessed the mice using NSS, NOR and MWM after injury. First, we found that mice treated with DA showed improved motor skills compared with those treated by vehicle (Fig. 1E). Nest, we conducted a NOR test to further evaluate memory retention. DA-treated mice spent significantly more time in exploring the novel object than the vehicle-treated mice (Fig. 1F). For MWM test, mice from different groups showed no differences in the hidden- or visible-platform trial performance (Fig. 1G and H). However, we observed a significant improvement during the probe test in the CCI + DA group compared with the CCI + vehicle group (Fig. 1I and J). Taken together, these data suggested that DA administration resulted in a possible reduction of neural death and restored some functional outcomes after CCI.

### DA inhibits neural death via DRD1

**Fig. 2:**
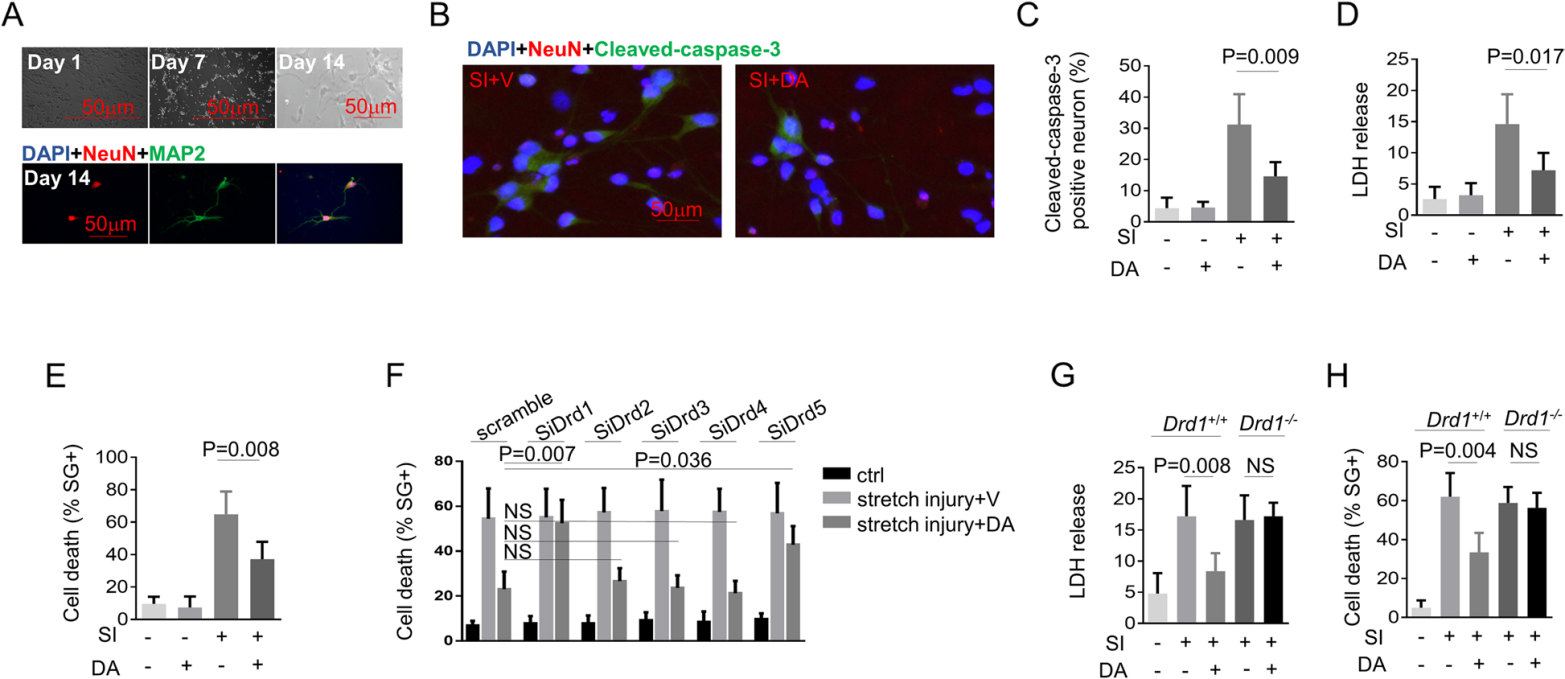
DA inhibits neural death via DRD1 in the model of stretch injury. **A** Representative photomicrographs of NeuN (red) and Map2 (green) expressing primary cortical neurons utilized in this study. **B** and **C** Primary neurons were subjected to stretch injury and then treated with DA (0.2mM) for 3 h. Representative photomicrographs of NeuN (red) and cleaved-caspase-3 (green) (*n*  = 3/group). **D** and **E** Primary neurons were subjected to stretch injury and then treated with DA (0.2mM) for 3 h. LDH release (**D**) and cell death (**E**) were measured by LDH assay and SytoxGreen positivity, respectively (*n* = 5/group). **F** Quantification of cell death from primary neurons which were transfected with control siRNA with a scrambled sequence or Drd1-Drd5 -specific siRNA as indicated, subjected to stretch injury and then treated for 3 h with DA (0.2mM). Cell death was measured by SytoxGreen positivity (*n *= 5/group). **G** and **H** Primary neurons from Drd1 *+/+* and D rd1 *−/−* mice were subjected to stretch injury and then treated for 3 h with DA (0.2mM). LDH release (**G**) and quantification of cell death (**H**) were measured by LDH assay and SytoxGreen positivity, respectively (*n *= 5/group). Data were represented as mean ± SD. NS, no significance

To confirm the effect of DA on TBI, primary neurons were subjected to SI using an in vitro TBI model (Fig. 2A). As expected, DA significantly suppressed the neural death after injury (Fig. 2B–E). We next explored the specific mechanisms underlying the inhibitory activity of DA on apoptosis. There are at least five subtypes of DA receptors that have been identified, termed DRD1–DRD5, and DA exerts its effects by binding to these receptors located on cell surface. To determine which receptor was functional under our experimental conditions, we silenced each of these receptors with small interfering RNAs (siRNAs), respectively (Supplementary Fig. 1). Our results showed that *Drd1* knockdown or knockout significantly suppressed the inhibitory effect of DA on SI-induced apoptosis, while knockdown of *Drd2*, *Drd3*, or *Drd4* displayed no such effect (Fig. 2F–H). In addition, *Drd5* knockdown even exerted a slight effect on DA-induced anti-apoptosis (Fig. 2F). These results suggested that DA inhibits SI-induced apoptosis primarily through DRD1. To further confirm this effect of DA, we obtained *Drd1*^*−/−*^ neurons from transgenic mice, and our data suggested that the inhibitory effect of DA on SI-induced apoptosis in primary neurons was partially or completely suppressed when *Drd1* was knocked out (Fig. 2G and H). Collectively, these results indicated that DA signals through DRD1 to inhibit SI-induced neural death.

### DA inhibits neural death via a RIPK1-dependent pathway

**Fig. 3:**
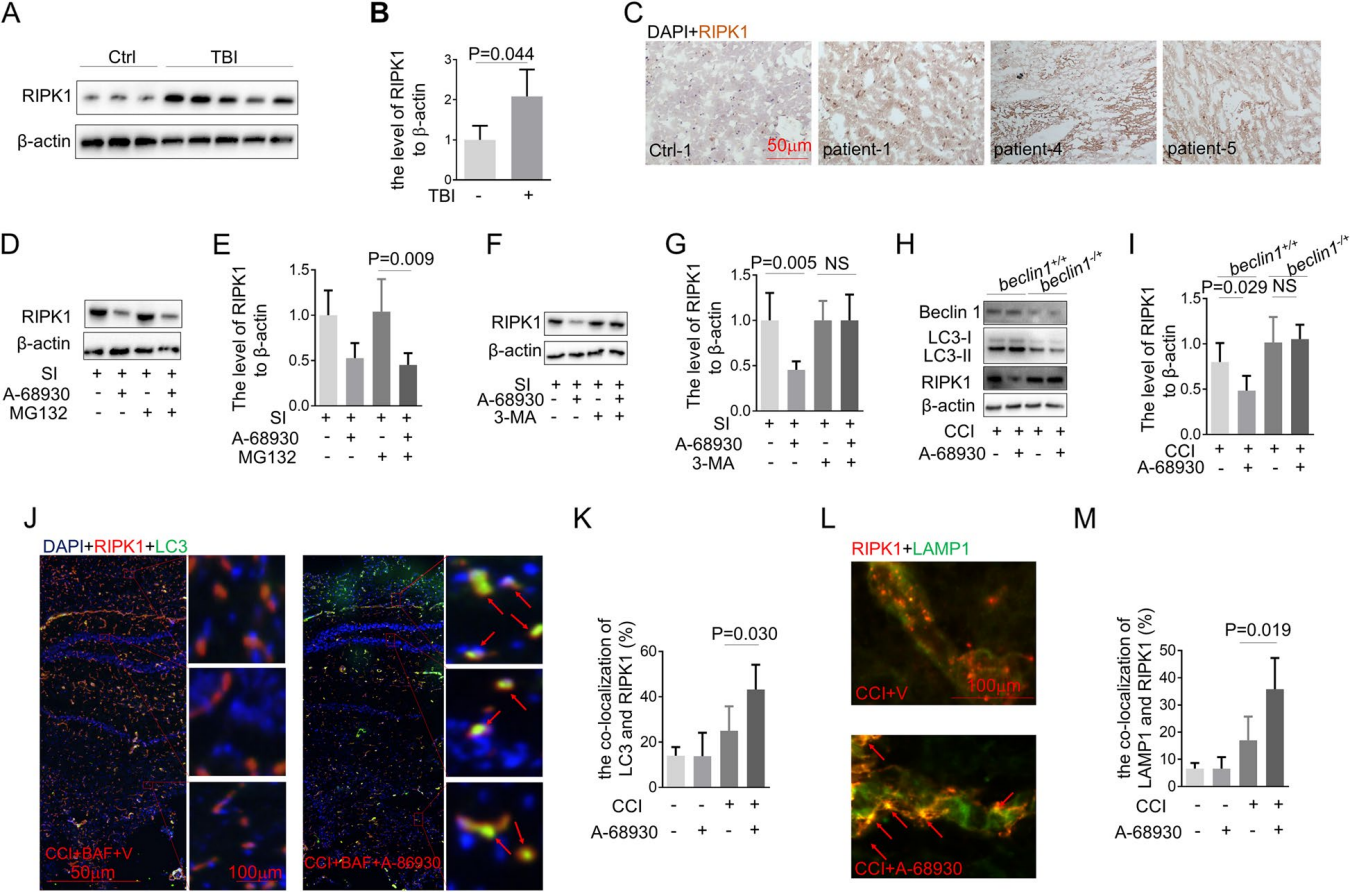
DRD1 signaling promotes RIPK1 degradation via autophagy. **A**-**C** Samples from patients with severe TBI were analyzed. The level RIPK1 was determined by immunoblotting (**A** and **B**) and immunohistochemistry (**C**). **D** and **E** Immunoblot analysis of RIPK1 and b-actin in cell lysates from primary neurons treated with MG132 (20μM) for 30 min and then stimulated with A-68930 (20μM) for 3 h (*n*=5/group). **F** and **G** Immunoblot analysis of RIPK1 and b-actin in cell lysates from primary neurons treated with 3-MA (2mM) for 30 min and then stimulated with A-68930 (20μM) for 3 h (*n*=5/group). **H** and **I** Immunoblot analysis of LC3, RIPK1, and b-actin in primary neurons from *beclin1*^-/+^ mice treated with A-86930 (20μM) for 3 h (n=5/group). **J** and **K** Immunofluorescence of LC3 (green) and RIPK1 (red) in brain tissue from mice, which were pre-treated by BAF (*n*=5/group). **L** and **M** Immunofluorescence of LAMP1 (green) and RIPK1 (red) in brain tissue from mice (*n*=5/group)

RIPK1 is a critical regulator of cell death and inflammation [16–18]. Contusional brain tissue resected from severe TBI patients was compared to brain tissue from control patients. Our data showed increased RIPK1 in TBI vs. control brain tissue (Fig. 3A-C). To gain further insights, we utilized in vitro mechanical stretch model and CCI model of in vivo TBI. A 68,930 is a potent and selective DRD1 agonist. Importantly, we found that A-68,930 treatment promoted RIPK1 degradation (Fig. 3D-G). Next, we investigated whether the degradation of RIPK1 protein was mediated by proteasome activity or autophagy, and our data signified that the proteasomal inhibitor MG132 could not suppress the RIPK1 degradation (Fig. 3D and E). 3-MA is one of the most widely used inhibitors of the initial phase of the autophagic process. We found that both the 3-MA and beclin1 deficiency significantly rescued A-68,930-induced RIPK1 degradation (Fig. 3F-I). BAF was known to prevent autophagosome–lysosome fusion. Moreover, A-68,930-treated mice showed more co-localization of GFP-LC3 with RIPK1 in the brain tissue in comparison to mice treated by vehicle (Fig. 3J and K). In addition, the co-localization of RIPK1 with lysosomes, detected using LAMP1, increased by A-68,930 treatment (Fig. 3L and M). These data suggested that the RIPK1 formed aggregates that were targeted for degradation by autophagy in our models.

### DRD1 signaling promotes RIPK1 ubiquitination via chip

**Fig. 4:**
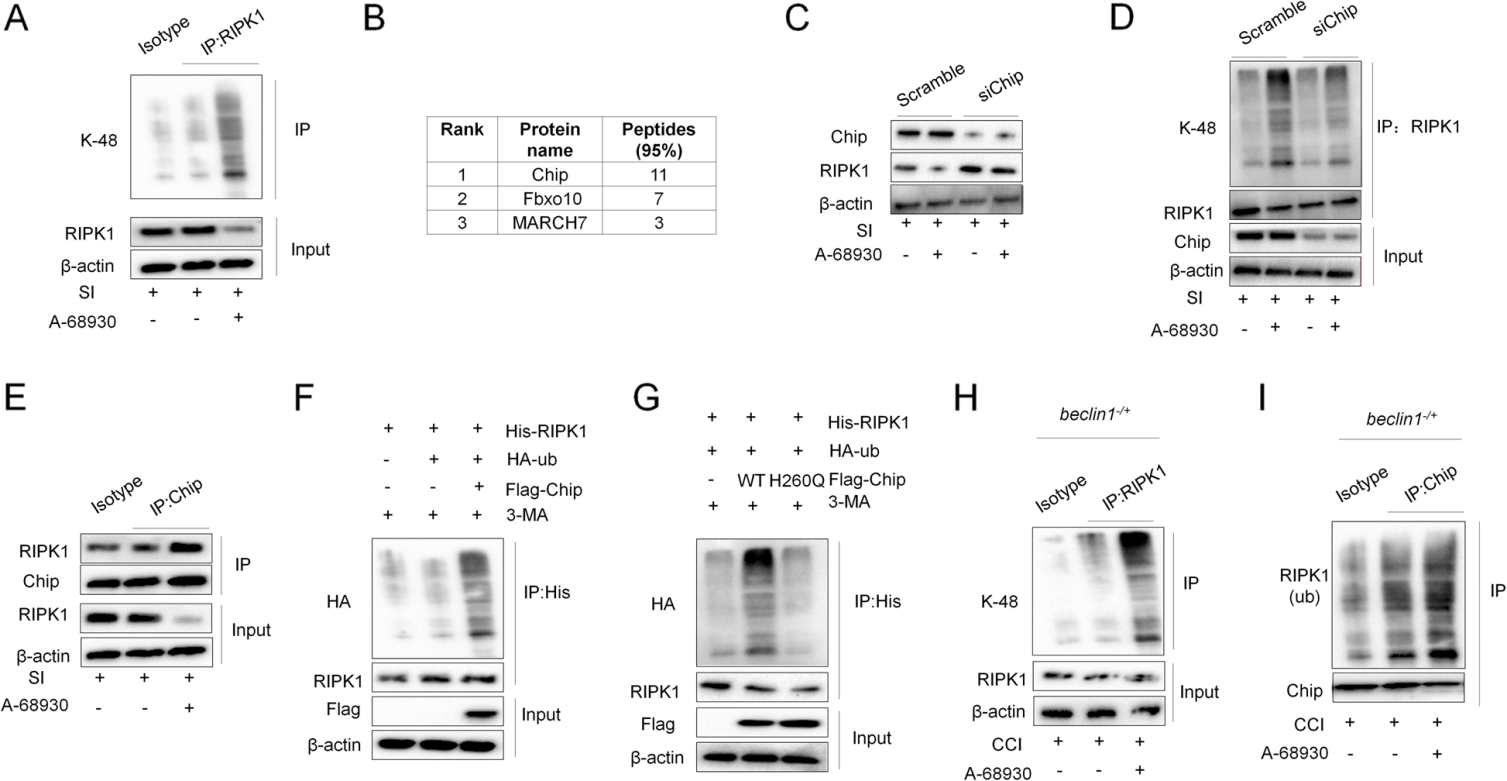
DRD1 signaling promotes RIPK1 ubiquitination and degradation via  Chip. **A** Primary neurons were subjected to SI and then treated with A-68930 (20 μM) for 1 h . Immunoblot analysis of K48-Ub in cell lysates immunoprecipitated with RIPK1 antibody (*n*=5/group). **B** List of E3 ligase proteins for top peptide hits identified in mass spectrometry study. (*n*=5/group). **C** Immunoblot analysis of RIPK1 and b-actin in cell lysates from A-68930-treated neurons transfected with siRNA against Chip (*n*=5/group). **D** Neurons were transfected with siRNA against Chip. After 48 h, the cells were subjected to SI and then treated with A-68930 (20 μM) for 1 h. Immunoblot analysis of K48-Ub protein from the cell lysates immunoprecipitated with anti-RIPK1 antibody (*n*=5/group). **E** Neurons were subjected to SI and then treated with A-68930 (20 μM) for 1 h. Immunoblot analysis of RIPK1 protein from the cell lysates immunoprecipitated with anti-Chip antibody (*n*=5/group). **F** Neurons were transfected with plasmids expressing HA–Ub, His–RIPK1 and Flag–Chip and then treated with 3-MA. The cell lysates were immunoprecipitated using anti-His antibody. RIPK1 ubiquitylation was detected by WB using anti-HA antibody (*n*=5/group). **G** Neurons were transfected with plasmids expressing with or without Flag–Chip or the Flag–Chip H260Q mutant. Immunoblot analysis of HA and RIPK1 from the cell lysates immunoprecipitated with anti-His antibody (n=5/group). **H ***Beclin1*^-/+^ mice were subjected to CCI. Immunoblot analysis of K48-Ub from the cortex lysates immunoprecipitated with anti-RIPK1 antibody (*n*=5/group). **I ***Beclin1*^-/+^ mice were subjected to CCI. Immunoblot analysis of RIPK1 from the cortex lysates immunoprecipitated with anti-Chip antibody (*n*=5/group)

We further demonstrated that A-68,930 induced a K48-linked polyubiquitination of RIPK1 in WT neurons that was also impaired by DRD1 deficiency (Fig. 4A) and next investigated which E3 ligase was responsible for A-68,930-induced RIPK1 ubiquitination and degradation. Mass spectrometric (MS) analysis was used to identify the E3 ligases associated with RIPK1 and showed that the E3 ligases Chip, Fbxo10, and March7 exhibited the greatest number of matched peptides identified by MS in the precipitates (Fig. 4B). Although the data revealed that knockdown of *Fbxo10* or *March7* using siRNAs did not affect A-68,930-induced RIPK1 degradation (Supplementary Fig. 2A and B), knockdown of *Chip* rescued A-68,930-induced RIPK1 degradation (Fig. 4C); furthermore. A-68,930-induced RIPK1 ubiquitination was partially inhibited by siChip (Fig. 4D). In addition, A-68,930 treatment also promoted an endogenous interaction between RIPK1 and Chip in the neurons (Fig. 4E). Ubiquitylation analyses under denaturing conditions indicated that Chip was able to induce polyubiquitylation of RIPK1, whereas H260Q exhibited an extremely reduced or abrogated ability to induce polyubiquitylation of RIPK1 (Fig. 4F and G). Moreover, A-68,930-treated *beclin1*^*−/+*^ mice showed more RIPK1 ubiquitination and interaction between Chip and RIPK1 in the brain tissue compared with mice treated by vehicle (Fig. 4H and I). These results collectively indicated that A-68,930 treatment induced RIPK1 ubiquitination and degradation via Chip.

### DRD1 signaling mitigates TBI-induced inflammation via an autophagy dependent pathway

**Fig. 5:**
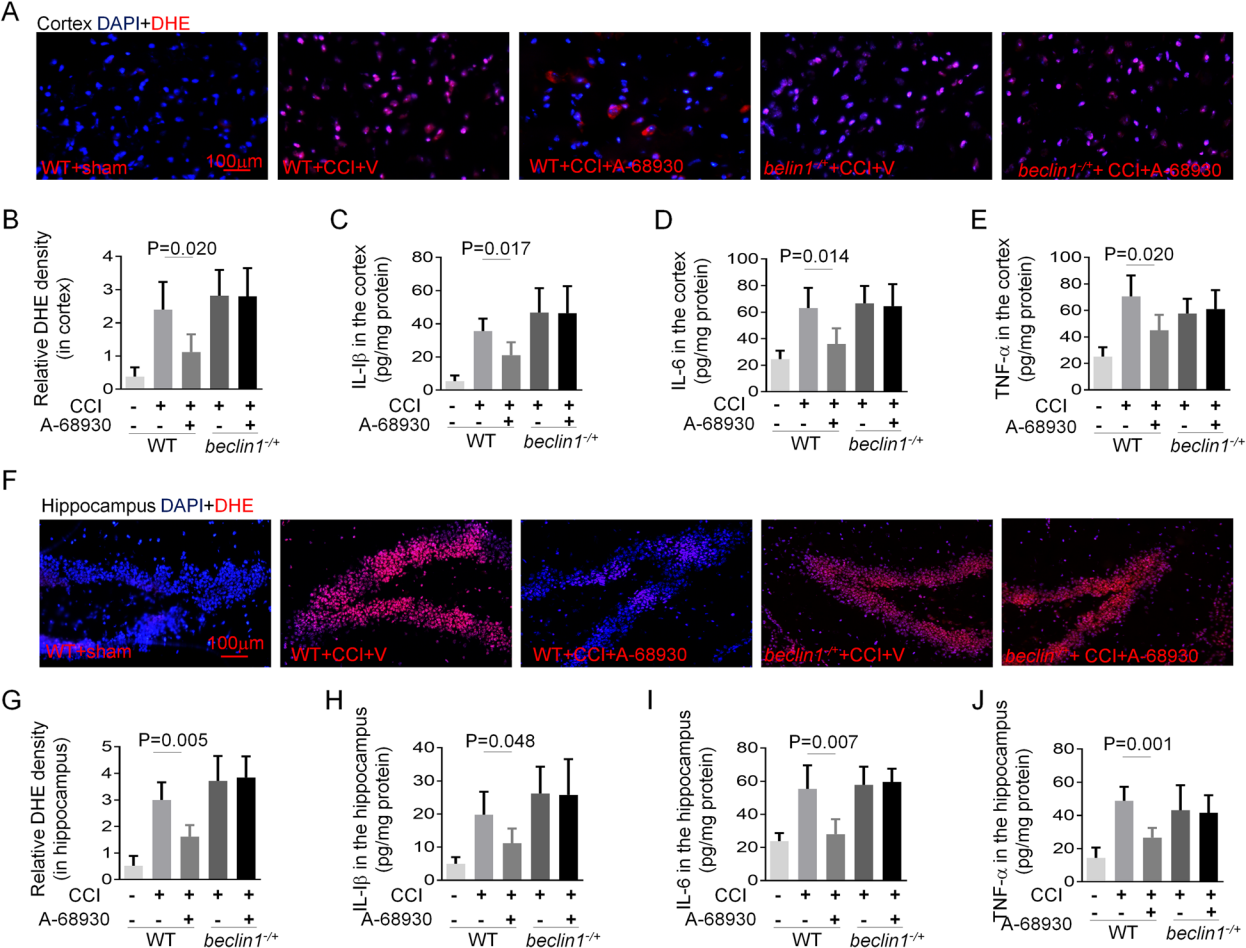
DRD1 signaling mitigates CCI-induced inflammation. **A** and **B** Representative images of DHE staining on the ipsilateral cortex, Scale bar, 100 µm (**A**). Quantifications of DHE fluorescence intensity on the ipsilateral cortex (**B**) (*n*=5/group). **C**-**E** Levels of inflammatory cytokines in the ipsilateral cortex. Levels of IL-1β (**C**), IL-6 (**D**) and TNF-α (**E**) in the cortex were determined by ELISA (*n*=5/group). **F** and **G** Representative images of DHE staining on the ipsilateral hippocampus, Scale bar, 100 µm (**F**). Quantifications of DHE fluorescence intensity on the ipsilateral hippocampus (**G**) (*n*=5/group). **H**-**J** Levels of inflammatory cytokines in the ipsilateral hippocampus. Levels of IL-1β (**H**), IL-6 (**I**) and TNF-α (**J**) in the hippocampus were determined by ELISA (*n*=5/group)

Since RIPK1 is a key mediator of multiple signaling pathways that regulate inflammation and apoptosis [16], we investigated whether A-68,930 treatment could suppress the TBI-induced neuroinflammation. ROS level is thought to contribute to neuroinflammation and assessed in the cortex or hippocampus using DHE staining. As expected, high levels of DHE signal in the cortex (Fig. 5A and B) and hippocampus (Fig. 5E and F) were significantly attenuated by A-68,930 treatment. However, ROS level in *beclin1*^*−/+*^ mice was not reduced by such treatment (Fig. 5A, B, E and F). Similarly, A-68,930 administration resulted in a remarkable reduction of IL-1β, IL-6, and TNF-α in both of the cortex (Fig. 5C-E) and hippocampus (Fig. 5H-J) of *beclin1*^*+/+*^ mice, but not of *beclin1*^*−/+*^ mice (Fig. 5). Taken together, these data suggest that A-68,930 mitigates TBI-induced inflammation via a beclin1-dependent pathway.

### DRD1 signaling mitigates CCI-induced cell death via an autophagy dependent pathway

**Fig. 6:**
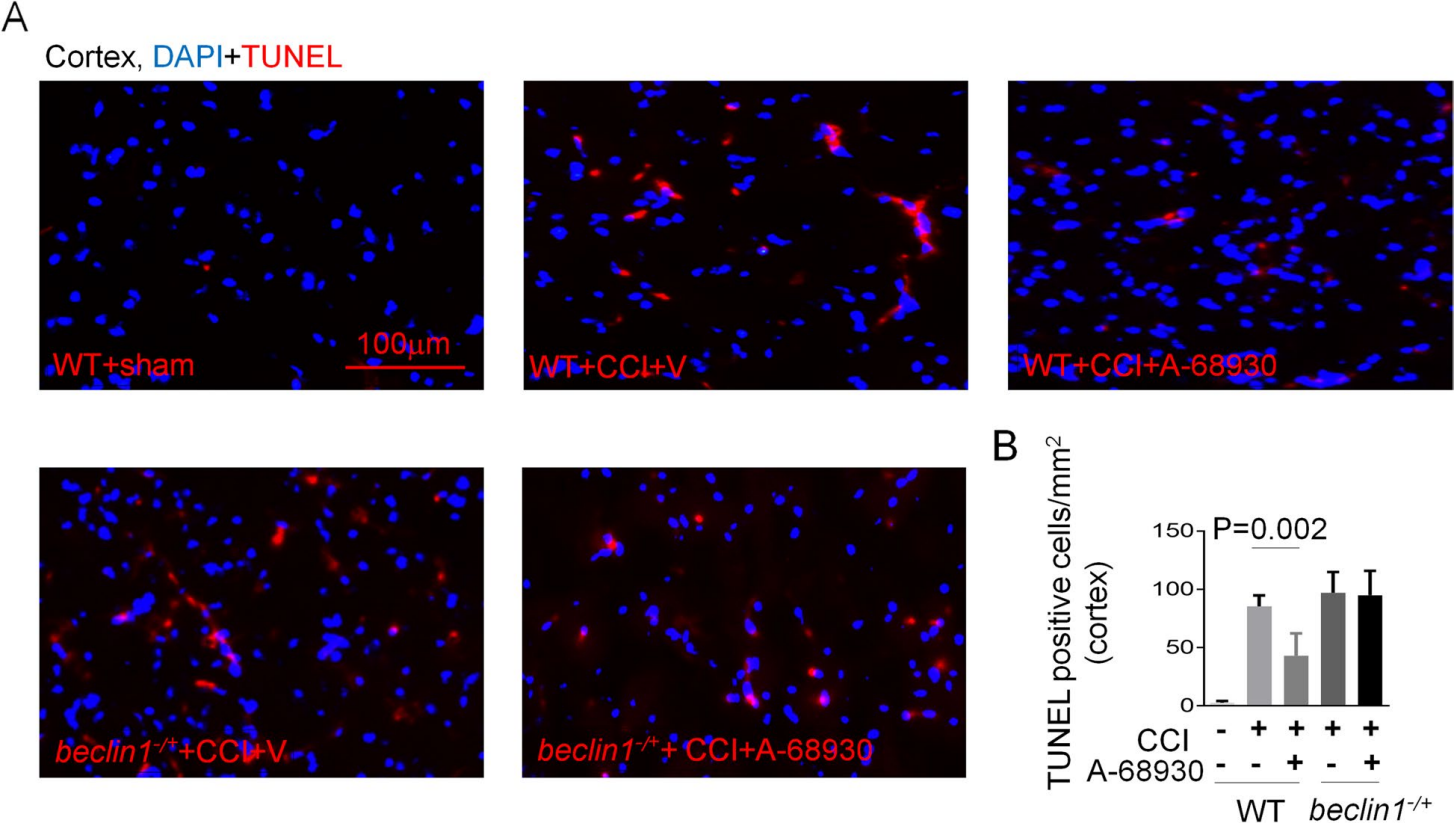
DRD1 signaling reduces CCI-induced cell death. **A** and **B** Representative fluorescence micrographs (**A**) and quantification (**B**) of the ipsilateral cortex after TBI. Apoptotic cell death was assessed by TUNEL staining (scale bar, 100 µm).* n*= 8/group

Compared with mice treated with vehicle, A-68,930 treatment reduced the number of TUNEL-positive cells in the cortex of *beclin1*^*+/+*^ mice after CCI, but not of *beclin1*^*−/+*^ mice (Fig. 6A and B). Taken together, these data above showed that A-68,930 treatment significantly prevented CCI-induced cell death via an autophagy-dependent pathway.

### DRD1 signaling rescues CCI-induced behavior deficits via an autophagy dependent pathway

**Fig. 7:**
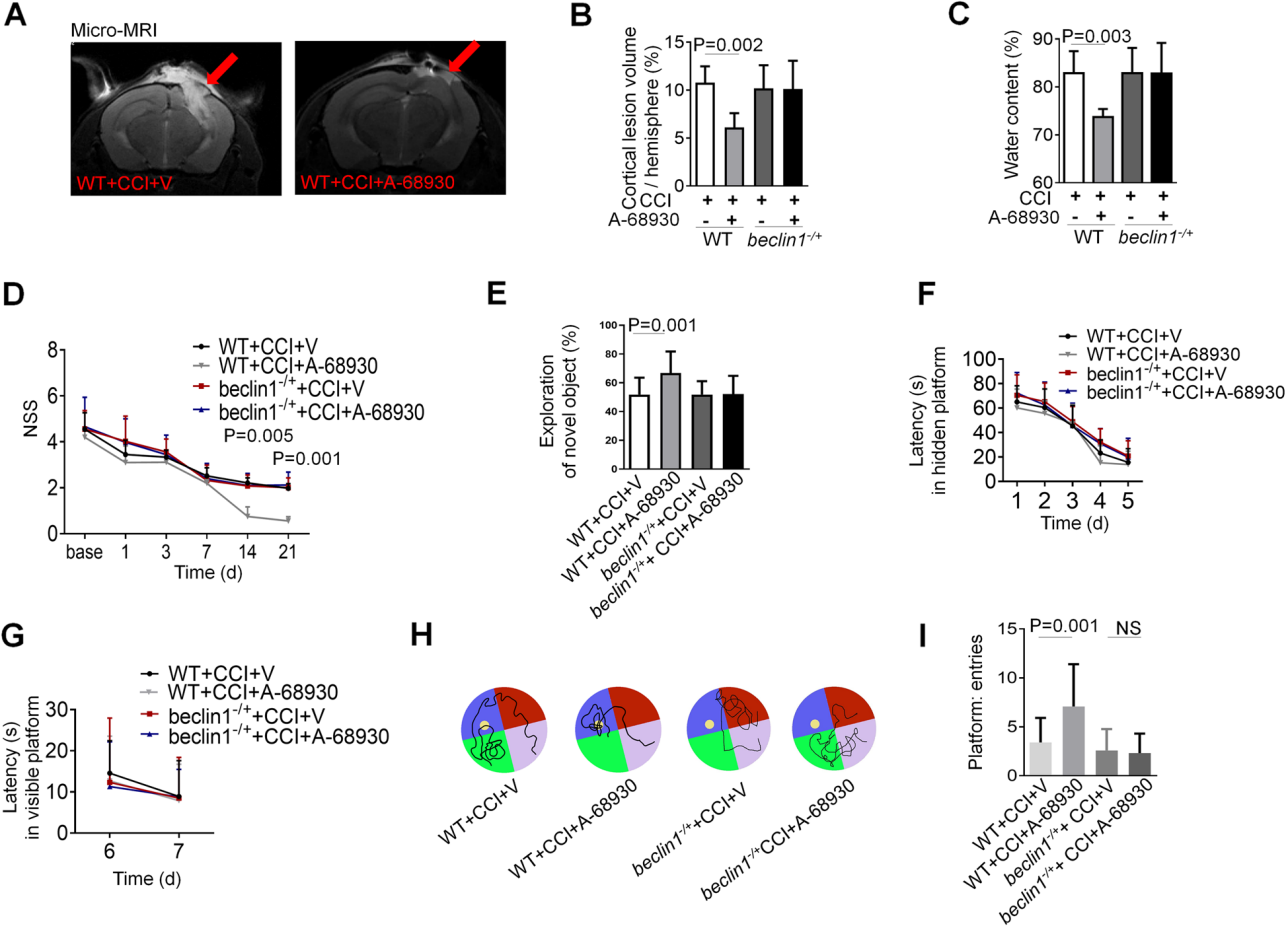
DRD1 signaling mitigates CCI-induced histological and functional deficits via an autophagy-dependent pathway. **A** Representative T2 weighted MR images showing lesion volume in mouse brain after CCI in different experimental groups. **B** Quantification of cortical lesion volume (n = 5 each group). **C** Analysis of water content (*n* = 5 each group). **D** Neurological severity score (NSS, between A-68930- and vehicle-treated WT mice). **E** Novel object recognition (NOR). **F**-**I** Morris water maze (MWM). Graph showing hidden platform trial (**E**) and visible platform trials (**F**). Representative images (**G**) and quantification (**H**) of probe tests. **D**-**I** WT+TBI+V mice, *n* = 20; WT+TBI+A-68930 mice, *n* = 25; *beclin1*^-/+^ +TBI+V mice, *n* = 25; *beclin1*^-/+^ +TBI+A-68930 mice, *n* = 25. All data are mean ± SD. V, vehicle. NS, no significance

We subsequently executed histologic and neurobehavioral tests to assess the neuroprotective potential of A-86,930 in the treatment of CCI. First, we found that cortical lesion volume was lessened in the A-68,930-treated mice relative to the mice that received vehicle (Fig. 7A and B). Furthermore, brain water content was significantly diminished in the CCI group treated with A-68,930 versus animals treated with vehicle, suggesting that A-68,930 had the effect on ameliorating brain edema induced by CCI. (Fig. 7C). However, A-68,930 treatment could not inhibit the CCI-induced histologic defects apparent in *beclin1*^*−/+*^ mice (Fig. 7A–C).

To further determine the impact of A-68,930 on behavioral and functional outcomes after CCI, we evaluated the mice using NSS, NOR, and MWM after injury. Mice treated with A-68,930 showed improved motor skills (Fig. 7D), as they spent significantly more time in exploring the novel object than did the vehicle-treated mice (Fig. 7E), and thus behaved similarly to the sham mice (Fig. 7D and E). In MWM, no differences were observed in the hidden- or visible-platform trial performance (Fig. 7F and G). We observed a significant improvement during the probe test in the CCI + A-68,930 group compared with the CCI + vehicle group (Fig. 7H and I). However, A-68,930 treatment could not rescue CCI-induced behavior deficits in *beclin1*^*−/+*^ mice (Fig. 7D–I).

## Discussion

DA not only functions as a neurotransmitter, but also functions as a local chemical messenger in the brain. Over several years, both clinical studies and animal experiments have demonstrated that DA exhibits anti-inflammatory activity and occupies beneficial roles in TBI [14, 19]. Thus, understanding the underlying anti-inflammatory and anti-apoptotic mechanisms would be of critical benefit in the appropriate use of DA in the clinic. There are five different DR subtypes, referred to as DRD1 to DRD5; these subtypes can be divided into two groups, D1-like receptors (including DRD1 and DRD5) and D2-like receptors (DRD2, DRD3, and DRD4) [14]. Although both groups are G-protein-coupled receptors and are activated by DA, they possess different signaling pathways. Their distinct positions may be explained by their diverse downstream transduction pathways. Previous results indicated that DRD1- and DRD2-signaling pathways exerted disparate roles in the suppression of neuroinflammation [14, 20]. However, DRD1 only functions to suppress inflammation under “stressed’’ conditions [11, 15]. Congruent with this, our study found that DRD1 signaling was critical to the inhibitory effect of DA on TBI-induced neuroinflammation and neural death. We demonstrated that A-68930, a specific agonist of DRD1, reduced acute cellular injury, brain tissue damage, motor dysfunction, and spatial-learning deficits after CCI in mice.

RIPK1 is an important mediator of multiple signaling pathways that are activated by members of the death receptor family and by pattern recognition receptors [17, 18]. RIPK1 kinase performs pro-cell-death and proinflammatory activities by activating RIPK1-dependent apoptosis and necroptosis [17], and emerging evidence suggests that RIPK1 also triggers severe inflammation by directly activating the inflammasome or inflammatory pathway [21, 22]. Increased RIPK1 expression in the human brains with TBI might be crucial not only as a mechanism that promotes neural death and neuroinflammation in the acute phase, but also sensitizes a variety of chronic traumatic encephalopathy, including Alzheimer’s disease and Parkinson’s disease [11, 23]. Our data support the concept that inactivity of RIPK1 reduces neuroinflammation and improves functional outcome after CCI in mice [24, 25]. The ubiquitin-proteasome system and autophagy are respectively responsible for approximately 80–90% and 10–20% of intracellular proteolysis [26–28]. Central to both degradative pathways is ubiquitination. The activation of RIPK1 is regulated by complex ubiquitin modifications [29, 30]. In our model, DA promoted RIPK1 ubiquitination via Chip and its degradation through autophagy. Ubiquitinated proteins generated on the surface of cargos are responsible for the recruitment of specific autophagy adaptor protein [15, 27]. Further analysis using a variety of disease models is required to broaden our understating of the link between chaperone-related lysosomal pathways and pathophysiologic disease states.

In conclusion, we demonstrated that DA and DRD1 signaling suppressed RIPK1 expression and identified an endogenous regulatory mechanism underlying RIPK1-dependent neural death after TBI. Our results also revealed that DA and DRD1 signaling prevented TBI-induced neuroinflammation. A clearer understanding of the role occupied by DRD1 signaling in RIPK1-mediated neural death may provide us with further insights into this signal-transduction mechanism, and also contribute to the development of drugs related to RIPK1-associated human diseases.

### Supplementary Information

**Additional file 1: Supplementary Table 1.** Clinical information of human brain specimens. **Supplemental fig 1.** Assessment of siRNA on Drd1, Drd2, Drd3, Drd4 and Drd5. **Supplemental fig 2.** Assessment of E3 ligase proteins.
