# Supplementary material for: Dopamine enhances recovery after traumatic brain injury through ubiquitylation and autophagic degradation of RIPK1
Source: Cell Commun Signal. 2024 Feb 19;22:134. doi: 10.1186/s12964-024-01515-y (PMC10875858; doi:10.1186/s12964-024-01515-y)
Supplement: Supplementary file 1 — Additional file 1: Supplementary Table 1. Clinical information of human brain specimens. Supplemental fig 1. Assessment of siRNA on Drd1, Drd2, Drd3, Drd4 and Drd5. Supplemental fig 2. Assessment of E3 ligase proteins. [file 12964_2024_1515_MOESM1_ESM.docx]

**Supplementary Table 1. Clinical information of human brain specimens.**

| No. of patient | gender | age | diagnosis | time to injury | site | Outcome |
| --- | --- | --- | --- | --- | --- | --- |
| Ctrl |  |  |  |  |  |  |
| 1 | male | 52 | epilepsy | — | right temporal lobe | no disability |
| 2 | female | 38 | epilepsy | — | left temporal lobe | no disability |
| 3 | male | 42 | epilepsy | — | left temporal lobe | no disability |
| TBI |  |  |  |  |  |  |
| 1 | male | 53 | TBI | 6 h | right frontal lobe | severe disability |
| 2 | female | 62 | TBI | 7 h | left temporal lobe | mild disability |
| 3 | male | 61 | TBI | 3 h | right frontal lobe | severe disability |
| 4 | female | 55 | TBI | 7 h | right temporal lobe | death |
| 5 | male | 48 | TBI | 4 h | left temporal lobe | mild disability |

Ctrl, control; TBI, traumatic brain injury; h, hour.


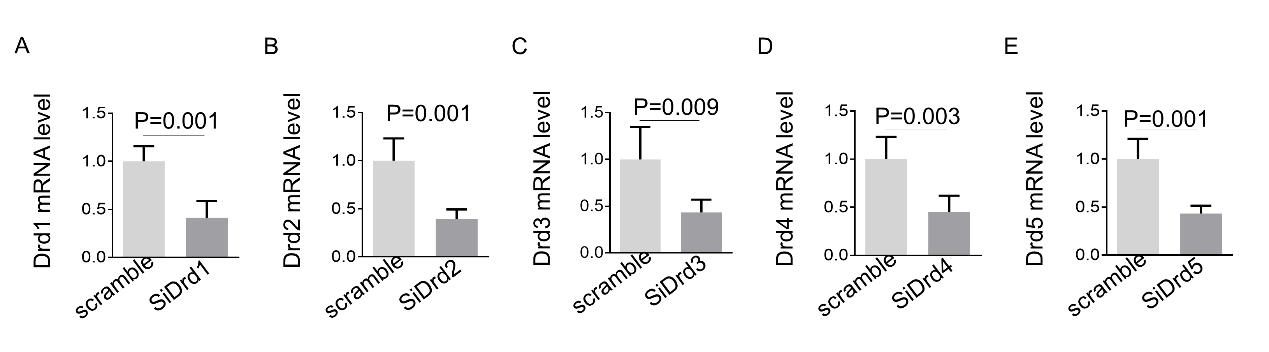


Supplemental fig 1. Assessment of siRNA on Drd1, Drd2, Drd3, Drd4 and Drd5.

(A) Quantification of Drd1 mRNA level .

(B) Quantification of Drd2 mRNA level .

(C) Quantification of Drd3 mRNA level .

(D) Quantification of Drd4 mRNA level .

(E) Quantification of Drd5 mRNA level .

Data presented as mean ± SD (n = 3/group).


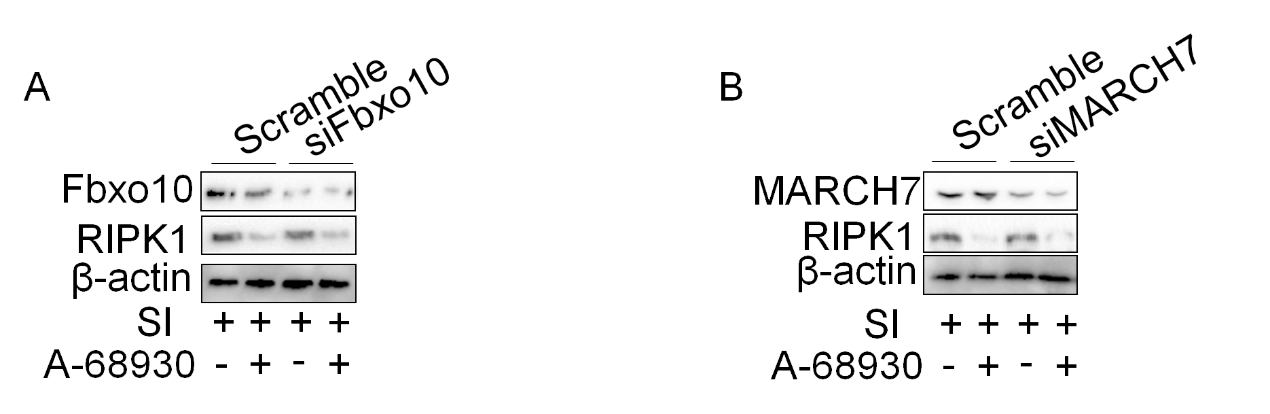


Supplemental fig 2. Assessment of E3 ligase proteins.

(A) Immunoblot analysis of RIPK1 and β-actin in cell lysates from A-68930-treated neurons transfected with siRNA against Fbxo10.

(B) Immunoblot analysis of RIPK1 and β-actin in cell lysates from A-68930-treated neurons transfected with siRNA against MARCH7.

Data presented as mean ± SD (n = 3/group).
